# Initial Development and Preliminary Validation of Khasi Word Lists for Speech Audiometry in Adults

**DOI:** 10.7759/cureus.102925

**Published:** 2026-02-03

**Authors:** Abhijeet Bhatia, Zareen A Lynrah, Manu Coimbatore Balakrishnan, Collette W Nongsiej, Larisa Kharmawphlang

**Affiliations:** 1 Otolaryngology - Head and Neck Surgery, North Eastern Indira Gandhi Regional Institute of Health and Medical Sciences, Shillong, IND

**Keywords:** khasi language, speech audiometry, speech in noise, speech in quiet, word lists

## Abstract

Background: Speech audiometry requires linguistically and culturally appropriate test materials to accurately assess functional hearing ability. The present study aimed to develop and preliminarily validate equivalent Khasi word lists for adult speech audiometry in quiet and in noise conditions.

Methods: This descriptive, cross-sectional observational study was conducted in the otorhinolaryngology department of a tertiary care teaching hospital in Shillong, Meghalaya, India, from February 2025 to July 2025 on adult Khasi participants. Adult native Khasi speakers with normal hearing on pure tone audiometry were recruited at each stage after obtaining written informed consent. An initial pool of 200 commonly used Khasi words (145 monosyllabic, 55 bisyllabic; consonant-vowel-consonant (CVC) and consonant-vowel-consonant-vowel (CVCV)) was identified from literature, dictionaries, and media. Ten native speakers rated word familiarity on a five‑point Likert scale, and 80 words (40 monosyllabic, 40 bisyllabic) with scores ≥4 were shortlisted. Comprehensibility (equality) testing was carried out in 10 normal‑hearing adults at a 20 decibel (dB) sensation level, and words correctly identified by ≥70% of participants were retained. Four lists of 20 words each (10 monosyllabic, 10 bisyllabic) were formed. List equivalence in quiet was assessed in five normal‑hearing adults at 40 dB sound pressure level (SPL) using live stimuli presented via a calibrated clinical audiometer in a sound‑treated room; word recognition scores (WRS) were analyzed using repeated‑measures analysis of variance (ANOVA). The same lists were then evaluated in another five adults in noise at signal‑to‑noise ratios (SNRs) of −3, −5, and −10 dB using speech‑shaped noise, and WRS were analyzed similarly.

Results: The four lists showed high mean WRS in quiet (range = 18.6-19.8/20), with no statistically significant difference between lists (p = 0.1081). In noise, there were no significant differences in mean WRS between lists at any SNR (−3 dB: p = 0.3425; −5 dB: p = 0.577; −10 dB: p = 1.00), and all lists maintained high scores across SNRs. Descriptive phonemic analysis demonstrated comparable distribution of consonants and vowels across the four lists.

Conclusion: Four preliminarily validated and equivalent word lists in Khasi have been developed for adult speech audiometry. These lists are suitable for use in both quiet and noisy environments at varying SNRs. These lists fill an important gap in regional audiological assessment tools and can be used clinically for evaluating speech recognition and monitoring outcomes of hearing rehabilitation among Khasi‑speaking adults, while further validation in larger and hearing‑impaired cohorts is warranted.

## Introduction

Khasi belongs to the Austro‑Asiatic language family and is indigenous to the Khasi Hills region of the Indian state of Meghalaya. In the 2011 census, approximately 1.4 million speakers reported Khasi as their mother tongue in India, and sizeable communities exist in neighboring Bangladesh. Khasi is written in the Latin script and has four principal dialects: Bhoi, Pnar, Lyngngam, and War. In clinical and educational settings, a standardized variety based on the central Khasi dialect is commonly used [1,2].

Speech audiometry provides information about functional hearing performance beyond pure tone thresholds, including speech discrimination in quiet and noise and the benefit from interventions such as hearing aids and cochlear implants. Because speech audiometry is language‑dependent, test materials must be developed and validated in the listener’s native or most familiar language to avoid underestimation of performance and false‑negative results. The Khasi‑speaking population constitutes around 47% of the population of Meghalaya, and a large proportion of patients attending the authors’ institution are native Khasi speakers who may not be fluent in English or Hindi. To date, no standardized Khasi speech audiometry material has been published, and clinical practice often relies on non‑native language lists, which may compromise diagnostic accuracy and rehabilitation planning [3,4].

Several languages have developed phonetically or phonemically balanced word and sentence materials for speech audiometry, following systematic steps including word selection, familiarity rating, intelligibility (equality) testing, list formation, and psychometric evaluation in quiet and in noise conditions [5,6]. These include Kannada, Tamil, Marathi, Ilocano, French, and Spanish materials, among others [7-9].

Objectives

The primary aim of this study was to develop and validate Khasi speech audiometry materials. The specific objectives were (1) to develop phonetically balanced Khasi word lists for adult speech audiometry in quiet, and (2) to evaluate the equivalence of these lists across quiet and multiple signal-to-noise ratios (SNR) in noise.

## Materials and methods

This descriptive, cross‑sectional observational study was conducted in the department of otorhinolaryngology of a tertiary care teaching hospital in Shillong, Meghalaya, India, after obtaining Institutional Ethics Committee clearance on 31.01.2025 with reference no. NEIGR/IEC/M5/F20/2024. The study was conducted over a period of six months from February 2025 to July 2025. All procedures adhered to the principles of the Declaration of Helsinki. Adult participants (≥18 years) who reported Khasi as their first language and demonstrated normal hearing thresholds (≤25 decibel hearing level (dB HL) at octave frequencies from 250 to 4000 Hz) on pure tone audiometry with normal tympanometry were included at each stage after obtaining written informed consent. dB HL is the universal reference scale for pure-tone thresholds in clinical audiology. It is calibrated so that 0 dB HL = average normal hearing threshold across speech frequencies, with normal hearing defined as ≤25 dB HL [5]. This being a pilot study and due to feasibility constraints, a total of 30 participants, with a distribution of five to 10 participants, at various stages of the study, were recruited. Participants with a history of otologic disease, otologic surgery, neurologic disorders affecting hearing or speech, or significant exposure to other dialects/languages as their primary home language were excluded. No participant took part in more than one stage of the study. Participants were recruited from among hospital staff and attendants/relatives of patients attending the otorhinolaryngology outpatient department, ensuring representation from different socio‑economic strata.

All testing was performed in a double‑walled sound‑treated room meeting American National Standards Institute (ANSI) standards for permissible ambient noise levels of 35 dBA (A-weighted decibel). Stimuli were presented via a calibrated clinical two‑channel audiometer (GSI Audiostar Pro GS 005769, Grason-Stadler, Eden Prairie, MN) using telephonics dynamic headphone (TDH) type supra‑aural earphones calibrated according to current ANSI/International Electrotechnical Commission (IEC) standards. Speech stimuli were administered live by a native Khasi‑speaking female adult of standard central Khasi dialect using a high‑quality condenser microphone at a fixed mouth‑to‑microphone distance, and normalized to a consistent root‑mean‑square level. Standard speech‑shaped noise from the clinical audiometer was used for speech‑in‑noise testing, in conjunction with live‑voice presentation of the Khasi word lists. Calibration of speech and noise levels, as well as SNRs, was verified using a sound level meter and the audiometer’s volume unit (VU) meter before each test session.

Selection of words

An initial corpus of 200 commonly used Khasi words was compiled in consultation with native speakers, language teachers, and published sources (Khasi literature, school textbooks, dictionaries, and local media programs) to ensure broad familiarity. Of these, 145 were monosyllabic consonant-vowel-consonant (CVC) words, and 55 were bisyllabic consonant-vowel-consonant-vowel (CVCV) words. Candidate words with taboo or ambiguous meanings or strong regional/dialectal restrictions were excluded at this stage based on expert consensus.

Familiarity assessment

The 10 native Khasi speakers who rated familiarity had a mean age of 29.55 years (range = 18-50 years); six were female, and four were male. They independently rated the familiarity of each of the 200 words using a five‑point Likert scale (1 = unfamiliar, 5 = very familiar). For each word, an overall familiarity score was obtained by averaging the individual ratings. Words with an average rating of less than 4 from any rater were excluded to ensure high and consistent familiarity. The selected words were shortlisted by two experienced audiologists based on content validity (clarity of pronunciation, lack of homophones, and clinical suitability). The use of familiarity ratings to refine word lists follows established procedures in the development of speech audiometry materials in Kannada, Marathi, and other languages [7-9].

Comprehensibility (equality) testing

The shortlisted 80 words were then subjected to comprehensibility or equality testing to ensure that they were of similar difficulty. Ten normal‑hearing adult Khasi speakers (not involved in the familiarity assessment) with a mean age of 33.4 years, age range of 22-47 years, comprising five males and five females, listened to the words presented monaurally at a 20 decibel sensation level (dB SL) relative to each participant’s speech reception threshold (SRT). A level of 20 dB SL was chosen for comprehensibility testing to provide clearly audible suprathreshold presentation in normal‑hearing adults, as commonly used in equality/intelligibility checks during speech‑test development in other languages like Kannada and Ilocano, wherein 20 dB SL was used [7-9]. Sensation level thresholds are personalized (threshold + 20 dB), and this approach ensures that differences in word recognition at this level reflect inherent word difficulty rather than audibility limitations, consistent with methodological precedents for establishing comparable item difficulty in test development [7-9]. Participants were instructed to repeat each word exactly as heard, and responses were scored dichotomously as correct (1) or incorrect/no response (0) by a trained audiologist. No formal inter-rater or intra-rater reliability checks were performed; however, scoring followed strict dichotomous criteria. Words correctly recognized by at least 70% of participants were considered adequately comprehensible and were retained for list construction. This equality‑testing step, though not universal, has been recommended in the preparation of speech audiometry materials in several languages to enhance uniformity of word difficulty [7-9].

Construction of word lists

All retained words met the comprehensibility criterion and were randomly allocated to four lists of 20 words each, with each list containing 10 monosyllabic and 10 bisyllabic words. Randomization was constrained to achieve approximate phonemic balance across lists, such that the distribution of initial and final consonants and vowel categories was broadly comparable.

Standardization of word lists in quiet

For evaluation in quiet, the four lists were administered to a new group of five normal‑hearing adult Khasi speakers with a mean age of 36.8 years, an age range of 25-46 years, and five females, in the sound‑treated room. For standardization in quiet, 40 decibel sound pressure level (dB SPL) was selected as a comfortable suprathreshold level in normal‑hearing adults, in line with previous word‑ and sentence‑list development studies that assess list equivalence at fixed suprathreshold levels in quiet [7,8,10-13]. This is an absolute physical sound pressure of 40 decibels (measured against a 20 micro-Pascal air pressure reference), equivalent to comfortable conversational speech, used for quiet standardization because it is a fixed, reproducible level across all participants/sessions, representing typical clinical testing conditions [7,8]. Selected word lists were presented monaurally at a fixed level of 40 dB SPL. The order of list presentation was randomized across participants to minimize order and fatigue effects. Participants were instructed to repeat each word, and responses were scored as correct (1) or incorrect/no response (0) by a single experienced examiner; ambiguous productions were clarified immediately and scored conservatively as incorrect if exact repetition was not achieved. No formal inter-rater or intra-rater reliability checks were performed; however, scoring followed strict dichotomous criteria.

For each participant and each list, a word recognition score (WRS) was calculated as: WRS (%) = (number of correctly repeated words / 20) × 100. The mean WRS and standard deviations (SD) for each list in quiet were then computed. Differences in mean WRS among the four lists were assessed using repeated‑measures analysis of variance (ANOVA), with list as the within‑subject factor. Data were visually screened for normality and sphericity; standard Greenhouse-Geisser corrections were applied as a conservative measure given the small sample size (n = 5). A p‑value <0.05 was considered statistically significant.

Standardization of word lists in noise

For evaluation in noise, the same selected lists were presented in the presence of calibrated speech‑shaped noise at three SNRs (−3, −5, and −10 dB) following the standardization protocol established for Kannada phonemically balanced word lists and sentence identification materials [7,8]. These negative SNRs indicate that the noise level is higher than the speech level by 3, 5, or 10 dB. They were selected to assess list equivalence across challenging listening conditions from moderate difficulty (-10 dB) while remaining within the performance capabilities of normal-hearing participants (-3 dB), consistent with the multi-SNR testing approach used in prior Indian language speech audiometry development studies [7,8]. The participants were an independent group of five normal‑hearing adult Khasi speakers with a mean age of 35.75 years, an age range of 20-42 years, comprising three males and two females, and none of these participants had taken part in the quiet condition. For each SNR, the speech level was fixed, and the noise level was adjusted to achieve the target SNR, verified by audiometer volume unit meters and sound level meter readings.

Each participant listened to all four lists at each of the three SNRs. The order of SNRs and lists was randomized and counterbalanced across participants to minimize learning and fatigue effects. Instructions, response recording, and scoring procedures were identical to the quiet condition. For each SNR, WRS (%) was computed for each list and participant, and the mean WRS with SD was derived. Repeated‑measures ANOVA was used to examine differences in mean WRS between lists at each SNR, and descriptive trends in WRS across SNRs were also described. Data were visually screened for normality and sphericity; standard Greenhouse-Geisser corrections were applied as a conservative measure given the small sample size (n = 5). The data were collected in Microsoft Excel 2017 (Microsoft Corporation, Redmond, WA), and data analysis was done using SPSS version 19.0 (IBM Corp., Armonk, NY).

## Results

In step 1, 200 words were selected. Out of the initial 200 words, 159 words met the familiarity criterion (average Likert rating ≥4) and were considered highly familiar to native Khasi speakers. From these, 80 words (40 monosyllabic and 40 bisyllabic) were selected after content validity review by audiologists in step 2. All 80 words were correctly recognized by at least 70% of participants during comprehensibility testing (step 3) at 20 dB SL and were therefore retained for list formation.

Following constrained randomization and phonemic balancing, four lists of 20 words each (10 monosyllabic, 10 bisyllabic) were finalized in step 4.

Performance in quiet (step 5)

During the standardization in quiet at 40 dB SPL, WRS were high across all lists for the five normal‑hearing participants. Mean WRS (±SD) for lists 1 to 4 were 19.8 ± 0.4, 19.0 ± 1.0, 19.6 ± 0.9, and 18.6 ± 1.1, respectively, out of a maximum of 20. Repeated‑measures ANOVA showed no statistically significant difference in mean WRS among the four lists in quiet (p = 0.1081), indicating that the lists were equivalent in terms of intelligibility under quiet listening conditions. Across all four lists in quiet, individual WRS ranged from 17/20 to 20/20, with an overall mean WRS of 19.3/20 (96.5%) for the 20‑word lists.

Performance in noise (step 6)

In noise, individual and mean WRS were calculated for each list at SNRs of −3, −5, and −10 dB. Mean WRS remained high at all SNRs, reflecting the relatively favorable listening conditions and the high familiarity of the material. At −3 dB SNR, repeated‑measures ANOVA showed no significant difference in mean WRS between lists (p = 0.3425); similar non‑significant results were obtained at −5 dB SNR (p = 0.577) and at −10 dB SNR (p = 1.00). An overall trend of decreasing WRS with lower SNRs was observed across lists, consistent with the expected psychometric behavior of speech‑in‑noise performance, although all lists maintained equivalently high scores at each SNR.

Table 1 summarizes mean ± SD WRS in quiet (40 dB SPL; n = 5) and in noise at −3, −5, and −10 dB SNR (independent speech‑in‑noise sample; n = 5).

**Table 1:** Word recognition scores in quiet conditions (40 dB SPL) and in noise (−3, −5, and −10 dB SNR) for four Khasi word lists. dB SPL: decibel sound pressure level; SD: standard deviation; SNR: signal-to-noise ratio. Quiet and noise conditions were obtained from two independent groups of five normal-hearing adults each (no participant overlap).

Word list	Performance in quiet (n = 5)	Performance in noise (n = 5)		
	Quiet 40 dB SPL (Mean ± SD)	−3 dB SNR (Mean ± SD)	−5 dB SNR (Mean ± SD)	−10 dB SNR (Mean ± SD)
List 1	19.8 ± 0.4	19.6 ± 0.6	20 ± 0	20 ± 0
List 2	19 ± 1	19 ± 1.2	20 ± 0	20 ± 0
List 3	19.6 ± 0.9	19.6 ± 0.5	19.8 ± 0.5	20 ± 0
List 4	18.6 ± 1.1	19.2 ± 1.3	19.6 ± 0.9	20 ± 0

In noise, individual WRS across all lists and SNRs ranged from 17/20 to 20/20, with overall mean WRS remaining above 95% at each SNR in this normal‑hearing sample. A ceiling effect was evident, particularly at −10 dB SNR (all lists: 20 ± 0), and near-ceiling scores across SNRs (overall >95%). The final lists are provided in Table 2.

**Table 2:** Final Khasi word lists for noise and quiet.

S. No.	List 1	List 2	List 3	List 4
1	jah	pan	rit	dor
2	wad	hep	jem	bor
3	mad	kot	meh	lum
4	jew	jot	sem	sada
5	kabu	suk	masi	pali
6	kali	mula	para	pela
7	dawa	kuli	pata	pura
8	jadu	duli	pule	ruma
9	lali	hati	risa	suba
10	sam	jaka	ruti	kam
11	paw	soh	bah	pep
12	maw	dap	rim	tur
13	rah	lap	tin	lok
14	duna	nala	tala	sur
15	hima	para	paro	suki
16	juti	paka	saja	suda
17	bam	rem	tah	dud
18	sor	tap	hok	dum
19	kubi	kada	kura	paka
20	saw	sati	riti	mula

## Discussion

This study describes the initial development and preliminary validation of four Khasi word lists for adult speech audiometry, following widely used methodological steps for creating language‑specific speech materials. The lists comprise familiar, phonetically balanced monosyllabic and bisyllabic words and have been shown to yield equivalent WRS in quiet and in noise at multiple SNRs among normal‑hearing adult Khasi speakers.

Historically, a wide range of speech materials has been used for audiological assessment, including nonsense syllables, isolated words, and sentences, each with specific advantages and limitations [6]. Nonsense syllables, while offering fine phonemic control, lack ecological validity and may not reflect real‑world speech perception [7]. Sentence‑based materials provide a more natural context but can be influenced by memory and linguistic redundancy, and often require longer administration time. Phonetically or phonemically balanced word lists offer a practical compromise and are now widely employed in clinical speech audiometry as they balance test efficiency and ecological relevance [8,9].

In the present study, word selection drew on sources of everyday language use to ensure high familiarity and face validity for lay speakers, consistent with prior work in Kannada, Marathi, and other languages [7-12]. Familiarity assessment using a five‑point Likert scale allowed objective elimination of less common words and is in line with procedures reported for Kannada phonemically balanced lists, Marathi open‑set tests, and Ilocano materials. Comprehensibility (equality) testing at 20 dB SL further ensured that the retained words were neither too easy nor too difficult to recognize when presented at a comfortable suprathreshold level, similar to the equality‑testing steps described in other language‑specific list developments.

Multiple lists are particularly valuable when repeated speech audiometry is needed, such as in monitoring hearing‑aid benefit or evaluating cochlear implant outcomes, as they help reduce learning and memorization effects [5]. For this purpose, the equivalence of lists is essential, so that different lists can be used interchangeably with similar expected performance. In this study, all four Khasi lists produced high and statistically indistinguishable WRS in quiet and at SNRs of −3, −5, and −10 dB, supporting their interchangeability under the tested conditions. The use of speech‑shaped noise and multiple SNRs aligns with contemporary speech‑in‑noise test development in French, Spanish, and other languages and facilitates clinically relevant assessment of suprathreshold speech perception in Khasi speakers [7-12]. The observed ceiling effects confirm high list intelligibility and equivalence in normal-hearing adults but suggest potential limited sensitivity for clinical populations with hearing impairment; more challenging (lower) SNRs or adaptive testing may be required.

The present work represents the first of its kind from the published literature of speech audiometry from Northeast India. The sample sizes at each stage, particularly during list standardization in quiet and in noise, were relatively small compared to some larger studies in Kannada and Tamil, which included tens to hundreds of participants [13]. This limitation restricts the precision of normative estimates and precludes detailed analyses of test-retest reliability and diagnostic accuracy. Table 3 shows the review of literature for speech audiometry in various other languages in a concise format.

**Table 3:** Review of literature for speech audiometry in various other languages. PB: phonetically balanced words; SNR: signal-to-noise ratio; SD: standard deviation; SIS: short increment sensitivity; WRS: word recognition score.

Author	Language, country	Type of material	Participants	Methodology in brief
Our study	Khasi language, India	PB words	Adults	Preparation of word lists - 200. Familiarity assessment- 159 words selected. Equality testing - 80 words selected. Construction of word list - 4 lists of 20 words each (10 monosyllabic and 10 bisyllabic words). Standardization of word list in quiet - comparison of mean with repeated measures ANOVA. Standardization of word list in noise - comparison of mean with repeated measures ANOVA.
Geetha et al. [7]	Kannada language, India	Sentences	Adults	Preparation of sentences - 700 sentences, with a mean of 4-5 words in each sentence. Naturalness testing - 10 participants. Predictability testing - 10 participants. 564 sentences were selected. Determination of global SNR - 8 participants. Equivalency testing - 15 participants. 316 sentences selected - phonemically balanced 30 lists with 10 sentences each selected. Standardization of sentence list - 100 participants. Comparison of the mean and SD of sentences repeated correctly.
Manjula et al. [8]	Kannada language, India	PB words	Adults	Preparation of word lists - 1200 bisyllabic words. Familiarity assessment - 15 participants. Equality testing - 820 words to 20 participants. Standardization of words in quiet - 65 participants. Administration of words in quiet - 65 participants. Standardization of words in noise - 100 participants. Administration of words in noise - 100 participants. Comparison of the mean WRS of words in quiet and noise. Final word lists - 21 lists with 25 words in each group.
Sagon [9]	Ilocano language, Philippines	PB words	Adults	889 words (bisyllabic and monosyllabic) - selected, 188 bisyllabic words - selected. Familiarity assessment done with 15 participants; two sets of 186 words, i.e., 372 words. 208 words selected, divided into 4 lists. Randomization - 3 word lists created with 50 words each.
Menon and Thangaraj [13]	Tamil language, India	PB words	Adults	Preparation of word lists - 500 bisyllabic words. Familiarity testing - 40 participants. Validation - 320 words to 8 experts. 270 words were chosen. Final word lists - 4 lists with 25 words in each group. SIS calculated for 120 normal-hearing participants. SIS calculated for 130 participants with hearing impairment. Comparison of SIS for normal and hearing-impaired participants.
Leclercq et al [11]	French language, France	Sentences	Adults	540 sentences (each 3-15 words), 127 sentences selected (each sentence of 7-11 syllables). Determining SNR-50 - 10 participants, assessment sentence difficulty - 12 participants, assessment of rapid speech in noise test - 3 list of 9 sentences, 29 hearing impaired participants.
Rodríguez-Ferreiro et al [12]	Spanish language, Spain	Sentences	Adults	240 sentences (each with 5 keywords). Familiarity assessment - 31 participants, 168 sentences - speech in silence and speech in Lombard effect - 30 participants. Equivalence was assessed, and SNR was calculated.

Future research should therefore evaluate these Khasi lists in larger samples of normal‑hearing adults across age groups, as well as in individuals with different types and degrees of hearing loss, to establish comprehensive norms, psychometric functions, and clinical cut‑offs. The use of monitored live‑voice presentation rather than standardized digital recordings was a limitation. While live‑voice testing allows for flexibility and is common in clinical practice, it introduces potential inter‑test and inter‑tester variability that can affect reproducibility. To minimize this, all stimuli were administered by a single trained native Khasi speaker, and presentation levels were strictly monitored using the audiometer’s VU meter. Future iterations of these word lists will be digitally recorded and standardized to eliminate speaker variability and ensure uniform presentation across different clinical settings. While strict scoring criteria were applied by an experienced examiner, future studies should incorporate dual independent scoring with reliability metrics to further strengthen the validation, given the absence of formal inter-rater or intra-rater reliability assessment for WRS scoring. While no statistically significant differences were observed between lists, formal equivalence testing was not conducted due to the preliminary nature of this validation study. Additionally, sensitivity to hearing‑aid fitting changes and performance in specific clinical populations (e.g., retrocochlear pathology) should be formally studied.

Despite these limitations, the present study demonstrates that it is feasible to develop culturally and linguistically appropriate speech audiometry materials for Khasi speakers using widely accepted methodological norms. These four preliminary lists can be incorporated into routine clinical practice to provide a more accurate assessment of speech recognition in native Khasi speakers, particularly for those who are not fluent in other test languages. In the longer term, the methods used here could be extended to create additional word lists and sentence or digit‑based materials to further enrich the Khasi speech audiometry test battery.

## Conclusions

Four phonetically balanced Khasi word lists, each comprising 20 highly familiar monosyllabic and bisyllabic words, were developed and preliminarily validated for adult speech audiometry in quiet and in noise at SNRs of −3, −5, and −10 dB. The lists demonstrated equivalent WRS across conditions in normal‑hearing adult Khasi speakers and are suitable for clinical use in this population, while further validation in larger and hearing‑impaired cohorts is warranted. In quiet conditions at 40 dB SPL, mean WRS across the four lists ranged from 93% to 99% with no statistically significant differences (p = 0.1081), confirming list equivalence under favorable listening conditions. In noise, mean WRS remained above 95% at all tested SNRs, with comparable performance across lists (p > 0.34 for all SNRs), supporting their reliability for assessing speech recognition in realistic background noise scenarios. These materials address a critical gap in audiological assessment tools for the Khasi‑speaking population of Meghalaya, enabling more accurate diagnosis and rehabilitation planning in routine clinical practice.
